# Community-Acquired Invasive GAS Disease among Native Americans, Arizona, USA, Winter 2013

**DOI:** 10.3201/eid2101.141148

**Published:** 2015-01

**Authors:** Aaron M. Harris, Del Yazzie, Ramona Antone-Nez, Gayle Dinè-Chacon, J.B. Kinlacheeny, David Foley, Seema Yasmin, Laura Adams, Eugene Livar, Andrew Terranella, Linda Yeager, Ken Komatsu, Chris Van Beneden, Gayle Langley

**Affiliations:** Centers for Disease Control and Prevention, Atlanta, Georgia, USA (A.M. Harris, S. Yasmin, L. Adams, C. Van Beneden, G. Langley);; Navajo Division of Health, Window Rock, Arizona, USA (D. Yazzie, R. Antone-Nez, G. Dinè-Chacon, J.B. Kinlacheeny, D. Foley);; Arizona Department of Health Services, Phoenix, Arizona (E. Livar, K. Komatsu);; Indian Health Service, Navajo Area, Arizona (A. Terranella, L. Yeager)

**Keywords:** group A streptococcus, GAS, invasive group A streptococcus, invasive GAS, Native American, necrotizing fasciitis, streptococcal toxic shock syndrome, outbreak, Arizona, bacteria

**To the Editor:** Group A streptococci (GAS) can cause severe invasive diseases, such as necrotizing fasciitis, streptococcal toxic shock syndrome, and sepsis. In 2012, ≈11,000 cases of invasive GAS (iGAS) disease and 1,100 associated deaths occurred in the United States (*1*,*2*). The risk for iGAS infection is 10 times higher among Native Americans than among the general population (*3*). Other predisposing factors for iGAS infection include skin wounds and underlying diseases, such as diabetes (*1*,*3*,*4*). Household risk factors include exposure to children with pharyngitis and crowding (*4*). Most iGAS infections occur sporadically within the community. Postpartum and postsurgical clusters arising from a common nosocomial source occur but are rare (*5*).

During the winter of 2012–13, a 3-fold increase in necrotizing fasciitis was observed at an Arizona hospital (hospital X) that predominantly treats Native Americans. Tribal leadership initiated a collaborative investigation with state and federal officials to characterize the outbreak and implement appropriate control measures.

A confirmed case of iGAS was defined as isolation of GAS from normally sterile sites (i.e., blood) or isolation of GAS from nonsterile sites (i.e., wound) in the presence of necrotizing fasciitis or streptococcal toxic shock syndrome among patients who sought care at hospital X during August 2012–March 2013. Hospital X serves ≈45,000 persons in a rural community. Eleven confirmed iGAS cases were identified (Figure), of which 8 (73%) occurred in women and 3 (27%) occurred in men. The case-patients had a mean age of 63 years (range 32–92 years). All cases were community-onset illnesses; none of the case-patients had recent exposures to health care settings, and all were of Native American ancestry. Of the 11 case-patients, 8 required critical care treatment and 3 died. Nine (82%) case-patients had open wounds or skin breakdown (e.g., skin abrasion, burns), and 9 had underlying medical conditions that are known risk factors for iGAS (e.g., obesity, diabetes, chronic kidney or heart disease, alcoholism).

**Figure:**
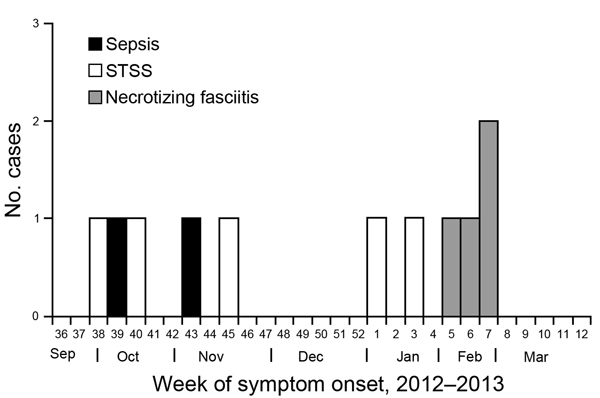
Week of symptom onset and principal clinical syndrome of patients with confirmed invasive group A streptococcus infections at hospital X, Arizona, August 2012–March 2013. STSS, streptococcal toxic shock syndrome.

Five GAS isolates were available. Two of the isolates were *emm* type 11; antimicrobial drug–susceptibility profiles for the 2 were identical (i.e., tetracycline resistant). The 2 patients reported no close contact with each other, but they had the same home health aide. The other 3 isolates had different *emm* types (1, 12, and 82) and were antimicrobial drug pansensitive.

We interviewed 58 household contacts of the case-patients (35 adults, 23 children) regarding symptoms and risks for secondary GAS infection. Among these contacts, 2 adults reported a sore throat and 6 children reported fever (without sore throat), but no confirmed secondary GAS infections were identified. Because of the known increased risk for iGAS among Native Americans and the level of crowding (average of 2–3 persons/bedroom) and the high proportion of adult household contacts with predisposing underlying conditions (29%) in this population, azithromycin prophylaxis was offered to household contacts who spent >24 hours with a case-patient during the 7 days preceding the onset of illness.

With the exception of the 2 case-patients with a common health aide, we found no common epidemiologic links or common behaviors among patients that suggested a single-source outbreak. This was further supported by the finding of multiple *emm* types among the isolates. These are not unusual findings in community outbreaks of iGAS; clusters of iGAS cases have often been observed without a common source (*6*–*8*). Localized and transient increases in sporadic GAS infections may occur because of an influx of a new *emm* type into a population with low levels of community immunity to that specific *emm* type; an increase in the detection and reporting of iGAS without a true increase in infection; or an increase in conditions that predispose persons to iGAS, such as GAS pharyngitis among children or concurrent influenza or other virus outbreaks in the community.

Past studies have shown that the risk of secondary iGAS infection among household contacts of patients with iGAS disease is higher than that among the general population but still low (*5*). Although Centers for Disease Control and Prevention guidelines do not recommend routine chemoprophylaxis for household contacts of patients with iGAS infection, the guidelines state that providers may choose to offer antimicrobial drug prophylaxis to those household contacts at increased risk for iGAS infection (*5*). Because Native Americans have increased rates of iGAS disease, compared with those of the general population, and because households in this investigation were crowded and many contacts had predisposing underlying conditions, we recommended that household contacts receive prophylaxis if given within 30 days of the index case-patient’s illness (*5*). No additional cases were reported at least 3 months after the investigation and intervention.
